# Hepatokines and polycystic ovary syndrome: investigating the connection between the hepato-ovarian axis and disorders in glycolipid metabolism in PCOS: a narrative review

**DOI:** 10.3389/fendo.2026.1598164

**Published:** 2026-03-30

**Authors:** Rui Zhu, Xinzhe Wang, Jingyun Ma, Xiuwen Shi, Yizhu Zhang, Jing Jin, Huifang Zhou

**Affiliations:** 1 Department of Gynecology, Affiliated Hospital of Nanjing University of Chinese Medicine, Nanjing, China; 2 School of Integrative Medicine, Nanjing University of Chinese Medicine, Nanjing, China

**Keywords:** hepatokines, hepato-ovarian axis, metabolic dysfunction, nonalcoholic fatty liverdisease, polycystic ovary syndrome

## Abstract

Polycystic ovary syndrome (PCOS) is a prevalent endocrine and reproductive disorder affecting 11-13% of women worldwide. It is defined by key clinical signs like elevated androgen levels and infrequent ovulation. PCOS, the most common endocrine cause of infertility in women of reproductive age, is often linked to insulin resistance, obesity, and other metabolic issues. It is closely associated with imbalances in the metabolism of glucose and lipids. Crucially, PCOS interacts with disorders of glucose and lipid metabolism. These metabolic disorders are the main signs of PCOS and can further aggravate the condition. As a vital metabolic organ, the liver produces a range of functional secretory factors known as hepatokines, which are crucial for metabolic regulation. These hepatokines circulate and exert a “Distant crosstalk” effect, influencing processes like glucose uptake, fatty acid breakdown, liver glucose production, inflammation, and various other functions in peripheral tissues. A recent connection between PCOS and nonalcoholic fatty liver disease (NAFLD) has been established, with the hepato-ovarian axis hypothesis considered a possible mechanism. As a result, the liver plays a key role in PCOS and is closely linked to the metabolic disorders involving glycolipids that are often seen with the condition. This review presents an in-depth overview of hepatokines that affect PCOS and its associated glycolipid metabolic disorders, providing key insights into the hepato-ovarian axis.

## 1 Introduction

**Figure 1:**
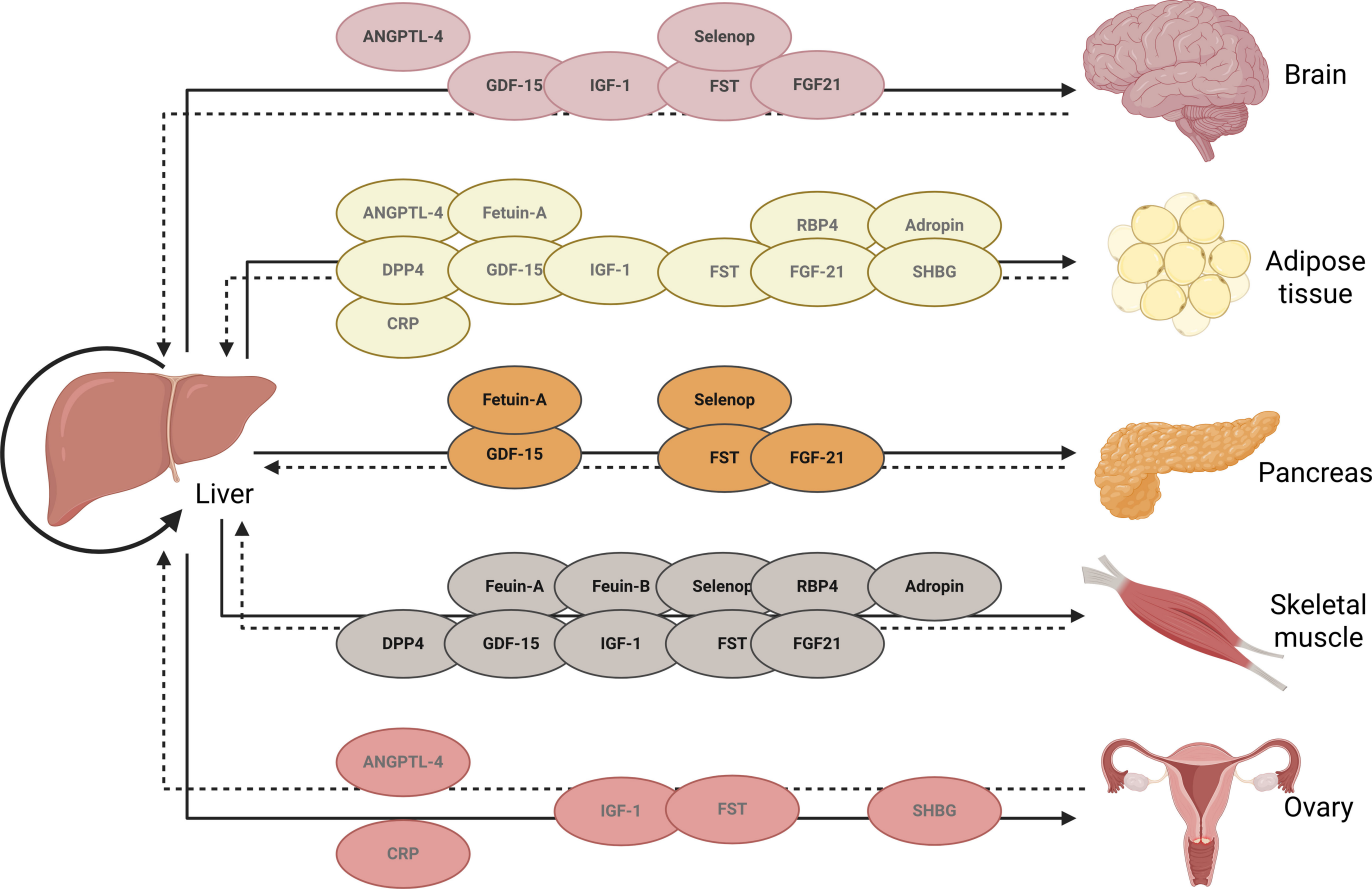
The liver “distant talk” with various organs by secreting hepatokines. The liver changes the physiology of the target organ by sensing various signals and plays a central role in metabolic activities. The liver communicates with multiple organs and tissues by producing hepatokines. It transmits metabolic information to target organs through hepatokines. This information is transmitted back to the liver to maintain metabolic balance in response to constant changes. ANGPTL-4, Angiopoietin Like 4; Selenop, Selenoprotein P; GDF-15, Growth differentiation factor; IGF-1, Insulin like growth factor-1; FST, Follistatin; FGF21, Fibroblast Growth Factor21; RBP4, Retinol-Binding Protein-4; DPP4, Dipeptidyl peptidase-4; SHBG, sex hormone-binding globulin; CRP, C-reactive protein.

Current clinical diagnosis mainly follows the criteria of the revised Rotterdam at 2023. This diagnosis requires at least two of the following three characteristics to be present: biochemical or clinical hyperandrogenism (HA), ovulatory dysfunction, and/or polycystic ovaries on ultrasound or elevated anti-Müllerian hormone (AMH) levels (1). The clinical manifestations of PCOS are highly diverse, affecting multiple body systems, and are primarily influenced by insulin resistance (IR) and obesity. These conditions are linked to the long-term metabolic outcomes of PCOS (2). Additionally, abnormalities in glycolipid metabolism within granulosa cells in PCOS impact oocyte development (3). The related metabolic abnormalities in glucose and lipids in PCOS have a detrimental effect on embryo quality and the success of early pregnancy (4). Consequently, the metabolic abnormalities related to glucose and lipid processing in PCOS have received more focus. IR affects 50%-70% of people with PCOS, irrespective of BMI (5). Abnormalities in blood glucose, lipid levels, obesity, and metabolic syndrome (MetS) are not only prevalent in PCOS, but also play a significant role in the long-term complications associated with the condition (6). Additionally, hepatic fat accumulation and HA have been linked to unfavorable metabolic risk profiles in PCOS (7). Around 25% of adults worldwide are impacted by NAFLD, which includes conditions from fatty liver (hepatic steatosis) to nonalcoholic steatohepatitis (NASH), with the potential to advance to serious liver complications like cirrhosis and even liver cancer (8). From a nomenclature perspective, NAFLD has recently been transitioned to metabolic dysfunction-associated steatotic liver disease (MAFLD) (9), while NASH has transitioned to metabolic dysfunction-associated steatohepatitis (MASH) (10). The prevalence of NAFLD/MAFLD in PCOS patients was significantly higher than that in healthy controls and was independently associated with homeostasis model assessment insulin resistence (HOMA-IR) and Alanine Aminotransferase (ALT). PCOS patients with overweight and elevated free androgen index (FAI) have a higher prevalence of fatty liver (11). (**Figure 1**) shows the liver “Distant talk” with various organs by secreting hepatokines).

**Figure 2:**
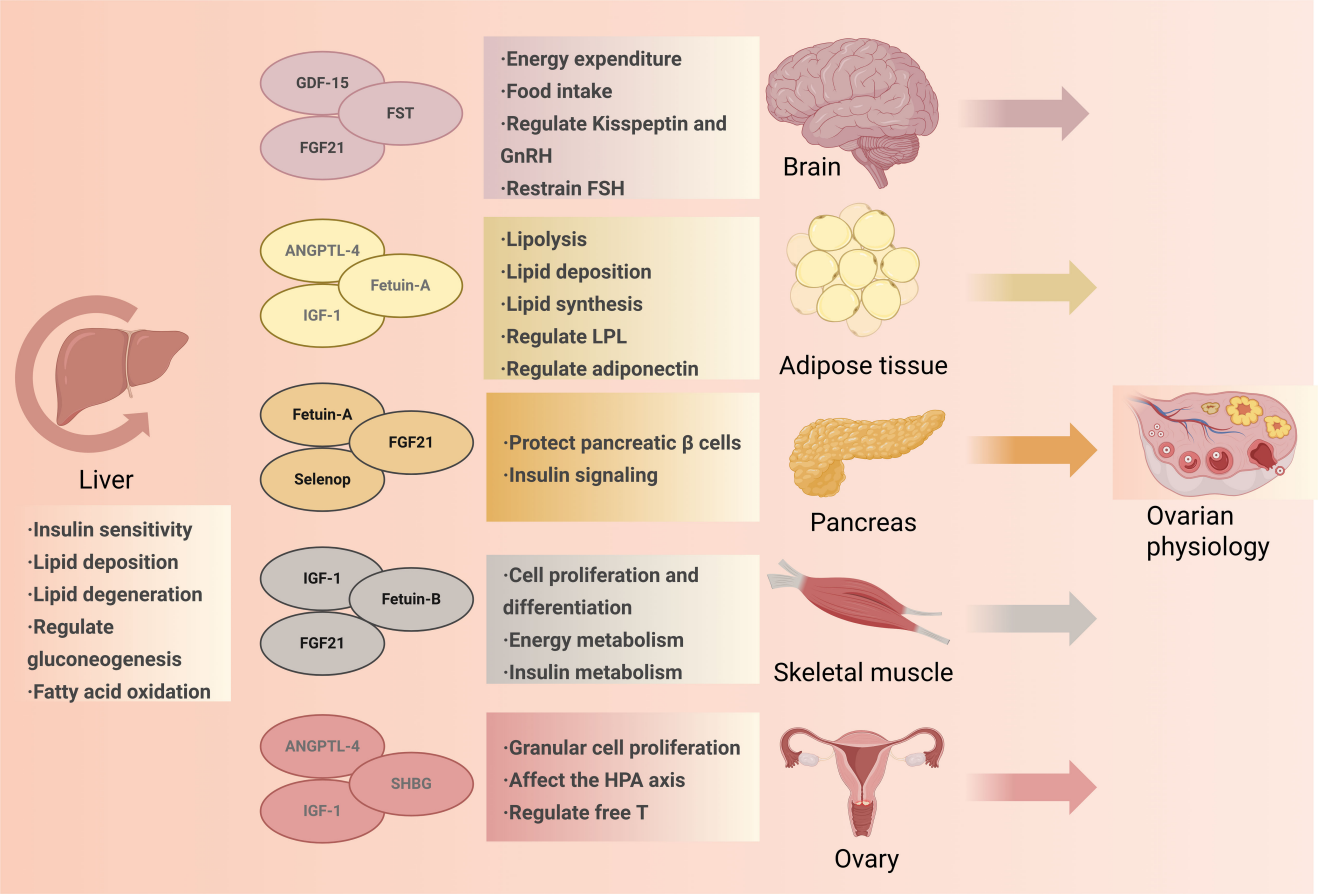
Physiological role of the liver on metabolism in different target tissues. Hepatokines have various effects on the whole body's metabolism and, finally, on the metabolism and endocrine of ovarian tissue. They regulate insulin sensitivity and lipid metabolism in the liver, energy metabolism, GnRH, and FSH in the brain, lipid metabolism in adipose tissue, protection of pancreatic beta cells, and insulin signaling in the pancreas. They also regulate energy metabolism, cell proliferation, and differentiation in skeletal muscle, affecting granulosa cell proliferation, the HPA axis, and serum-free testosterone concentration in the ovary. GDF-15, Growth differentiation factor; FST, Follistatin; FGF21, Fibroblast Growth Factor21; ANGPTL-4, Angiopoietin Like 4; IGF-1, Insulin like growth factor-1; Selenop, Selenoprotein P; RBP4, Retinol-Binding Protein-4; SHBG, sex hormone-binding globulin; GnRH, Gonadotropin-releasing hormone; FSH, follicle-stimulating hormone; HPA axis, The hypothalamic-pituitary-adrenal axis; T, Testosterone.

PCOS is strongly linked to NAFLD, with the risk of developing NAFLD in individuals with PCOS being 2–4 times greater than in the general population, regardless of body weight (12). As a result, NAFLD is more common and liver damage is more severe in individuals with PCOS. Among the NAFLD patients, 62% of them suffered from PCOS (13), and metabolic risk factors are more common in patients with PCOS (14). HA and IR are separate factors in the progression of NAFLD in individuals with PCOS (15). The underlying mechanism of the comorbidity of PCOS and fatty liver mainly attributes to IR and HA, and it also involves abnormal glycometabolism and dyslipidemia, obesity and chronic inflammation (16). Furthermore, there is a strong connection between PCOS and NAFLD in terms of metabolic disorders, which may potentially influence the progression and relationship between the two conditions. As the primary hub for metabolism, the liver helps maintain metabolic balance by interacting with other organs. Hepatokines are crucial to metabolic function throughout the body, and under conditions of heightened metabolic stress, their expression can be altered, potentially playing a role in the progression of PCOS, either on their own or in conjunction with other factors. The physiological and pathological changes of the liver can be transmitted to the hepato-ovarian axis by the hepatokines and form an interaction. Changes of hepatokines in PCOS and NAFLD lead to metabolic disorders (17, 18). In this paper, we comprehensively describe the expression of different hepatokines in PCOS and its related disorders of glycolipid metabolism to explore the possible mechanism of hepatokines’ crosstalk in PCOS and its complications. (**Figure 2** shows physiological role of the liver on metabolism in different target tissues).

## 2 Key hepatokines and their main physiological functions

### 2.1 ANGPTL-4

Angiopoietin-like 4 (Angptl-4) is primarily secreted by hepatocytes and adipose tissue, playing a crucial role in glycolipid metabolism. It acts as an important physiological regulator of lipoprotein lipase (LPL) (19). Angptl-4 also inhibits pancreatic lipase, an enzyme responsible for the hydrolysis of triglycerides (TG) (20) in the gastrointestinal tract, leading to increased adiposity and weight (21). It plays a crucial role in lipid distribution and homeostasis, reducing adipose tissue to restore fat balance while promoting the storage of ectopic fat (22), thus affecting glucose homeostasis. Angptl-4 significantly improves glucose tolerance, decreases blood glucose and induces hyperlipidemia and hepatic fat accumulation. However, Angptl-4 knockout mice failed to reveal the effects of Angptl-4 on glucose homeostasis (23), and existing studies disagree on the impact of Angptl-4 on glucose metabolism and insulin sensitivity (24). In tissues enriched with TG-rich lipoproteins and celiac particles, Angptl-4 reduces TG hydrolysis and prevents atherosclerosis. In macrophages, Angptl-4 reduces inflammation and apoptosis (25). Angptl-4 knockout mice exhibit reduced hepatic triglyceride accumulation and prevent the development of NAFLD (26). High expression of Angptl-4 plays a role in the development of PCOS by triggering phosphorylation in the EGFR/JAK1/STAT3 signaling pathway and preventing the proliferation of granulosa cells (27). Angptl-4 is markedly higher in individuals with PCOS regardless of body weight. The elevated concentration of Angptl-4 and its effects on metabolic pathways may play a role in the insulin resistance seen in PCOS (28).

### 2.2 Fetuin-A

The Fetuin family consists of Fetuin-A and Fetuin-B, which are primarily secreted by the liver. These proteins are paralogous homologs, sharing a similar amino acid sequence (29). Fetuin-A plays a key role in glucose and lipid metabolism and serves as a marker for metabolic diseases (30).

#### 2.2.1 In glucose metabolism

Fetuin-A is one of the important proteins in insulin-dependent metabolism, which is associated with impaired insulin sensitivity, ultimately leading to IR and its complications (1). Inhibit the insulin receptor tyrosine kinase, which leads to IR (31) (2). Participate IR indirectly by inducing lipid deposition in the adipose and liver, which causes inflammation (32) (3). Act as a marker for TLR-4 endogenous ligand. Free fatty acids stimulate adipose tissue inflammation and induce IR through the TLR-4 pathway, which is further enhanced by TLR-4 activation of the transcription factors NF-κB and activator protein-1 or Fos/Jun-induced production of inflammatory cytokines. Thus they enhance adipose tissue inflammation and IR (33) (4). Inhibit adiponectin and enhance adipose tissue IR by Wnt3a/PPARγ pathway (34) (5). Promote macrophage migration and conversion to M1 type in pancreatic β-cells, which triggers β-cell inflammation, impairs their functions, and aggravates IR (35). In addition, Fetuin-A impairs insulin sensitization and raises blood glucose levels leading to IR (36). Furthermore, Fetuin-A functions as an adipokine, with its expression directly related to the fat content in adipocytes, can subsequently enhance the absorption and storage of free fatty acids in these cells.

#### 2.2.2 In lipid metabolism

(1) Reduce adiponectin and impairs mitochondrial energy metabolism by inhibiting PPARγ phosphorylation (2). Enhance SREBP-1c by inducing mTOR phosphorylation, inducing adipogenesis and accumulation (34, 37), which collectively lead to hepatic steatosis (38).

#### 2.2.3 Regulating inflammation

Fetuin-A has shown pro-inflammatory effects in patients with IR, diabetes, obesity, atherosclerosis, MetS, NAFLD (38), and anti-inflammatory properties in systemic inflammation (39). In addition to this, Fetuin-A is an important inhibitor of vascular calcification and plays an important role in atherosclerosis and cardiovascular disease(CVD) (40). NAFLD is strongly linked to disruptions in glucose and lipid metabolism. Therefore, a substantial rise in Fetuin-A is closely related to ectopic fat in the liver (41). Elevated androgen levels enhance Fetuin-A expression via androgen receptors in hepatocytes (42), therefore, serum Fetuin-A levels are elevated in patients with PCOS (43).

### 2.3 Feuin-B

Fetuin-B, like Fetuin-A, is mainly produced in the liver (44). (i) In glucose metabolism, Fetuin-B inhibits glucose metabolism pathways by inhibiting the genes encoding glucose-6-phosphatase and phosphoenolpyruvate hydroxyacid kinase in hepatocytes. And it reduces insulin-mediated Akt phosphorylation, decreasing insulin sensitivity, and through non-insulin signaling pathway affecting glucose metabolism (45). (ii) In lipid metabolism, Fetuin-B inhibits lipid synthesis and promotes lipid metabolism. Knockdown of *FETUB* gene in rats significantly increased the level of fatty acid synthase, while the level of fatty acid metabolizing enzymes decreased, both lipid droplet formation and TG were significantly increased (46). In obesity, leptin-STAT3 activates the increased transcription of Fetuin-B in hepatocytes (47). Studies have shown that Fetuin-B links NAFLD to type 2 diabetes mellitus (T2DM) through IR (48). Proteomic analysis of follicular fluid in PCOS patients revealed a significant elevation of Fetuin-B, which is involved in biological processes related to inflammation, immune response, and metabolism. As a differentially expressed protein, Fetuin-B may serve as a potential biomarker and therapeutic target (49). In individuals with PCOS, Fetuin-B levels are substantially elevated in the serum and are independently associated with insulin resistance and liver fat accumulation, with these levels decreasing significantly as insulin resistance improves (50, 51).

Selenoprotein PSelenoprotein P(SeP)is mainly produced by the liver. (i) In glucose metabolism, the promoter of the Selenop gene is regulated by FoxO and PGC-1α, and is inhibited by insulin (52, 53). Serum SeP levels are elevated in T2DM and show a positive correlation with fasting glucose and HbA1c. Additionally, hepatic SePP1 mRNA levels are positively associated with fasting glucose. Both glucose clearance and metabolic clearance are positively correlated as well (54). (ii) In lipid metabolism, the expression of the *Sepp1* gene is decreased in obesity, likely due to the inhibition of SeP expression in 3T3-L1 adipocytes by pro-inflammatory and oxidative molecules (55). It is negatively correlated with adiponectin and high-density lipoprotein cholesterol (HDL-C) (56). It was also demonstrated that serum levels of SeP were significantly higher in individuals with NAFLD and abdominal obesity. The risk of NAFLD in those with elevated SeP was 6.5 times greater compared to those with lower SeP levels. Additionally, SeP was positively correlated with the liver attenuation index, HOMA-IR, and visceral fat area measured by computed tomography in NAFLD patients. Therefore, SeP could serve as a new serological diagnostic marker for NAFLD (56). Additionally, SeP has been found to have a negative correlation with MetS, total visceral and subcutaneous abdominal adipose tissue volume, hepatic signal intensity, and fatty liver disease (57). The level of SeP was significantly elevated in PCOS (58) and may be a biomarker of oxidative stress in it (59).

### 2.4 RBP4

Retinol binding protein 4(RBP4) is secreted primarily by the liver to transport vitamin A to target tissues (60), and secondarily by adipose tissue. It also acts as an adipokine that specifically binds to retinol, thereby linking obesity and insulin resistance. (i) In glucose metabolism (1), RBP4 decreases insulin sensitivity by inhibiting the insulin receptor substrate-1 (IRS-1) phosphorylation and phosphatidylinositol 3-kinase activation, at the same time, it simultaneously increases hepatic glucose production by increasing PEPCK expression (61) (2). RBP4 modulates retinol to alter tissue metabolism by increasing production of retinoic acid isoform, and can also cause IR by a retinol-independent mechanism (61). Although RBP4 levels may correlate with the extent of IR, it is controversial in adults with obesity and/or IR (62). RBP4 may be elevated in impaired glucose tolerance and T2DM by preventing thyroid hormone-binding proteins from binding to the receptor (63, 64). Additionally, overexpression of RBP4 triggers inflammation in adipose tissue by activating both the innate and adaptive immune responses, which in turn promote IR (65). RBP4 abnormalities precede elevated glucose in overweight/obese populations and correlate with BMI and IR. Thus, in obesity and/or T2DM, RBP4 may reflect improved insulin sensitivity by triggering IL-1β release in a glucose-dependent manner via the TLR4/MD2 receptor complex and TLR2, which indirectly inducing IR in adipocytes (66, 67). RBP4 is independently associated with MetS (68). It can also serve as a potential biomarker for NAFLD and indicate the severity (69). The specific mechanism involves hepatic cell-derived exosome RBP4, which converts Kupffer cells into the M1 subtype by mediating the NOX2/ROS/NF-κB pathway. This process then promotes adipogenesis in hepatocytes through the secretion of tumour necrosis factor-alpha (TNF-α), which further activates the JAK2/STAT3 signaling pathway. These events lead to the upregulation of RBP4 transcription (70), creating a vicious cycle. Elevated levels of RBP4 in PCOS are not associated with IR, but correlate with elevated levels of follicle-stimulating hormone (FSH) and luteinizing hormone(LH) (71). It has also been demonstrated that RBP4 levels in PCOS are negatively correlated with serum insulin levels and HOMA-IR values, while positively correlated with BMI, waist-to-hip ratio (WHR), and body fat percentage. Therefore, RBP4 might serve as a compensatory mechanism to prevent the worsening of obesity related PCOS (72).

### 2.5 Adropin

Adropin, primarily produced by the liver and brain, helps regulate glucose and lipid balance by influencing the expression of adipogenic genes in the liver and the peroxisome proliferator-activated receptor γ in adipose tissue (73). (i) In glucose metabolism, Adropin promotes insulin-induced Akt phosphorylation, which enhances insulin sensitivity. And it upregulates the expression of glucose transporter protein 1, which promotes glucose uptake by hepatic cells and improves IR (74, 75). (ii) In lipid metabolism, Adropin inhibits hepatic steatosis (73) and activates the Nrf2 pathway to trigger an antioxidant response to protect against hepatic injury in NASH (76). Serum Adopin was significantly negatively correlated with fasting glucose and insulin levels, which were significantly reduced in T2DM and IR (77). Adropin could serve as a potential indicator for predicting the risk of T2DM. Adropin levels were found to be negatively correlated with BMI, meaning that lower levels of Adropin are associated with obesity (78). In PCOS, reduced Adropin may be associated with elevated levels of TNF-α (79). Serum and follicular fluid adropin levels were positively associated with BMI and HDL-C levels, while negatively associated with LDL-C level (80). Reduced serum Adropin may be closely associated with abnormal branched-chain amino acid metabolism in the ovary, which influences the development of IR in PCOS. And Adropin may mediate HA by affecting the concentration of SHBG. Therefore, it may serve as a strong predictor of the long-term risk of MetS in patients with PCOS (81).

### 2.6 DPP4

The liver is an important source of circulating dipeptidyl peptidase 4 (DPP4) in the body (82). In glucose metabolism (1), DPP4 is directly involved in insulin production through inactivation of glucagon-like peptide. DPP4 inhibitors inhibit glucose production by decreasing DPP4 degradation, prolonging degradation of glucagon-like peptide 1 and inhibiting glucagon (83) (2). DPP4 increases glucose-dependent glucagon-releasing polypeptide and promotes insulin secretion and glucose uptake by peripheral tissues, thereby lowering blood glucose and reducing body weight (83). DPP4 levels are elevated in obese patients, and even more in those with obesity and IR, suggesting that it could serve as a marker for visceral obesity, IR, and MetS (84). Glucose tolerance was enhanced in mice lacking DPP4, which also prevented obesity and insulin resistance. Additionally, DPP4 inhibition reduced endothelial cell dysfunction and atherosclerosis in T2DM (85). In conclusion, hepatic DPP4 induces IR, reduces glycogen storage, and increases glucose output and lipid accumulation in the liver. Therefore, increased DPP4 expression may contribute to the development of NAFLD (86). Serum DPP4 activity and concentration are elevated in patients with PCOS, and its activity is strongly associated with AMH levels (87).

### 2.7 GDF15

The liver produces growth differentiation factor 15 (GDF15) in response to a high-fat diet or obesity, relaying peripheral metabolic signals to the brain to help adapt to energy demands during metabolic stress. GDF15 causes the death of pancreatic β-cells due to endoplasmic reticulum stress, and its deficiency prevents or delays the onset of diabetes mellitus in a mouse model (88). GDF15 helps reduce body weight and visceral fat by decreasing appetite and food intake (89). It could lower the risk of obesity, insulin resistance, and other related complications by boosting lipolysis and the metabolism of oxidized metabolites (90). In addition, GDF15 can reduce hepatic fat deposition and effectively improve NAFLD (91). Early-stage PCOS is strongly linked to a relative deficiency of GDF15 (92).

### 2.8 IGF-1

Insulin-like Growth Factor 1 (IGF-1) is primarily produced in the liver and regulated by growth hormone. Its activity is controlled by insulin-like growth factor binding proteins and the insulin-like growth factor-1 receptor (IGFR) (93). (i) In glucose metabolism, IGF-1 enhances glucose uptake and improves insulin sensitivity, (ii) In lipid metabolism, IGF-1 stimulates adipogenesis, promotes lipid uptake and oxidation, and reduces TG and cholesterol, thereby reducing adiposity (94). Obese patients often experience abnormalities in the growth hormone/IGF-1 axis, resulting in decreased levels of IGF-1. Additionally, obesity is commonly associated with IR, which triggers the release of numerous inflammatory factors. These inflammatory factors can also contribute to the reduction of IGF-1 level (95). Low serum IGF-1 correlates with the histologic severity of NAFLD (96), it also correlates with atherosclerosis, obesity and MetS risk (97). In the ovary, IGF-1 is produced by theca cells and follicular membrane cells, where it acts on the hypothalamic-pituitary-ovarian axis. It plays a crucial role in reproductive function, both independently and in combination with gonadotropins (98). IGF-1 acts as an important regulator in the ovary and influences the pathogenesis of PCOS (99) (1). IGF-1 overexpression results in downregulation of IGFR expression, increased responsiveness to LH sensitivity, resulting in dysfunction of the ovary and promoting PCOS (100, 101) (2). IGF-1 synergistically promotes the synthesis and secretion of androgen by insulin and LH, and it also synergistically further increases androgen secretion by affecting hepatic SHBG secretion, which indirectly increasing androgen bioavailability. IGF-1 and insulin work together to inhibit SHBG, thereby increasing free androgen levels (102). In conclusion, IGF-1 interacts with HI and HA in PCOS, and together play a role in the development of the condition. IGF-1 may be a crucial factor in the pathogenesis of PCOS (103).

### 2.9 Follistatin

In the liver, the ratio of glucagon to insulin regulates the secretion of cyclic Follistatin (Fst) (104). And Fst may has an influence on IR and inflammation by interacting with members of the TGF-β family (105). Fst could also function as an adipokine. In cases of obesity, the expression of Fst mRNA is decreased in subcutaneous adipose tissue. Additionally, Fst counteracts the growth inhibition that promotes adipogenic differentiation (106). Fst may play a role in mediating T2DM. In mice, Fst inhibits the breakdown of white adipose tissue by reducing insulin and IR levels in white adipose tissue. This results in an increase in free fatty acid levels, which contributes to the development of T2DM and NAFLD (107). Fst has the ability to inhibit the synthesis and secretion of FSH, also known as FSH-inhibitory protein (Fsp). Human follicular fluid contains a high concentration of Fsp, which is the primary source of Fst in the peripheral circulation. Fsp can directly block FSH secretion from anterior pituitary cells. Additionally, it can irreversibly bind to and deactivate activin-specific binding proteins, indirectly decreasing FSH secretion and, consequently, lowering estrogen levels in the body. Fsp is a crucial regulator of follicular development. Overexpression of the *FST* gene can lead to early infertility and arrest of follicular development, similar to conditions observed in PCOS (108). Circulating Fsp levels are significantly increased in PCOS and Fst levels are higher in obese PCOS. High Fst levels in PCOS may be related to its chronic low-grade inflammatory state (109).

### 2.10 FGF21

The liver is the main source of circulating fibroblast growth factor 21 (FGF21) (110) which primarily affects the liver, adipose tissue, and central nervous system. Initially, FGF21 functions as a hepatic energy regulator, playing a key role in the regulation of glucose and lipid metabolism.

#### 2.10.1 In glucose metabolism

(1) FGF21 increases glucose uptake by adipocytes and improves glucose metabolism and IR. FGF21 activates PPARγ in adipocytes and up-regulates the expression of adiponectin, which increases glucose uptake by adipocytes and reduces blood glucose levels. It also reduces blood glucose in an insulin-independent signaling pathway by up-regulating glucose transporter protein 1 in adipocytes (111) (2), FGF21 prevents cytokine-induced apoptosis in pancreatic islet β-cells (112) (3), It stimulates the downstream Ras-Raf-melanocyte-activated protein kinase signaling pathway, which induces peroxisome proliferator-activated receptor γ co activator-1a gene expression. This further activates the transcriptional activity of PPARγ, thereby regulating gluconeogenesis (113) (4), It promotes uptake of skeletal muscle glucose (114) (5), FGF21 also acts on the nervous system, such as①reducing glucose uptake by inhibiting the paraventricular nucleus of the hypothalamus (114), ② increasing glutamatergic neurons in the ventral medial hypothalamus to inhibit glucose uptake (115), ③ stimulating the hypothalamus by stimulation of adrenocorticotropic hormone-releasing hormone via ERK1/2-CREB signaling cascade, which in turn triggering the release of corticosterone to induce gluconeogenesis (116).

#### 2.10.2 In lipid metabolism

(1) It inhibits hepatic fat synthesis from scratch and suppresses the expression of the adipose transcription factor sterol regulatory element-binding protein 1c (117) (2), promotes hepatic fatty acid oxidation by (118) ① up-regulating the expression of long-chain lipoyl-coenzyme A synthetase and fatty acid transport proteins to activate fatty acids to acyl-CoAs, ② increasing the expression of PGC-1α and PPARα to promote fatty acid mitochondria expression, thereby promoting fatty acid mitochondrial oxidation (3), inhibiting hepatic very low-density lipoprotein receptors, thereby reducing hepatic TG accumulation (119) (4), promoting lipophagy in hepatic adipocytes to reduce fat deposition (120) (5), up-regulating the expression of heat-producing genes, such as uncoupling protein 1, in brown fat to promote the browning of white fat, which will regulate the whole-body lipid metabolism to improve NAFLD (121), or acts directly on brown adipose tissue and subcutaneous adipose tissue to enhance glucose uptake (122). Ameliorates NAFLD and NASH by ameliorating inflammation and stress injury (1): In terms of ameliorating inflammation: ① exerting anti-inflammatory effects by enhancing macrophage nuclear factor E2-related factor 2 and inhibiting the nuclear factor κB signaling pathway (123), ② inhibiting the recruitment of adiponectin-mediated helper T cell 17 and interleukin 17 secretion in a mouse model of NASH, thereby inhibiting the recruitment and activation of immune cells (124) (2). In terms of reducing oxidative stress injury: ① FGF21 can activate the phosphatidylinositol-3-kinase/protein kinase B signaling pathway, enhancing Nrf2-mediated antioxidant capacity and apoptosis (125). It can also activate the adenylate-activated protein kinase -silent information regulator 2-related enzyme 1 signaling pathway, which thereby enhancing mitochondrial activity and its oxidative capacity (126), ② FGF21 inhibits the eukaryotic initiation factor 2α-activating transcription factor 4 signaling pathway, thereby attenuating lipotoxicity and TG accumulation due to endoplasmic reticulum stress (127) (3). In terms of ameliorating fibrosis, FGF21 inhibits hepatic stellate cell (HSC) activation by down-regulating the expression of TGF-β, the phosphorylation of Smad2/3, the activation of NF-κB and the expression level of NF-κB inhibitory protein (IκBα), which in turn inhibit HSC activation, and these also increase the expression of cysteine protease 3 and decreases the expression of the B-lymphoblastoma 2 gene (Bcl-2)/Bcl-2-associated X protein ratio. All of these lead to apoptosis of activated HSC, thus inhibiting fibrosis (128). Moreover, FGF21 significantly down-regulated leptin signaling pathway-related proteins, at the same time, it was able to up-regulate upstream Nrf2 and cytokine signaling, negatively regulate the expression of factor 3 and ultimately blocked the platelet-derived growth factor-BBPDGF-BB-leptin axis to inhibit the activation of HSC (129).

#### 2.10.3 The relation between FGF21 and PCOS

FGF21 is an important regulator of lipids and glucose, which can reduce androgen levels and restore ovulation in PCOS patients by improving insulin sensitivity, promoting lipid metabolism and reducing body weight. The potential mechanisms by which FGF21 inhibits fertility, such as (1) regulation of the HPA or HPG axis by acting on downstream targets in the hypothalamus in the suprachiasmatic nucleus of the hypothalamus and hypothalamus Kisspeptin, as well as the pituitary gland. Higher level of FGF21 interferes with the liver-neuroendocrine axis by decreasing the expression of *Apv* and *Kiss1* genes in the anterior ventral periventricular nucleus nucleus, leading to a decrease in Kisspeptin. These lead to a surge in LH and abnormalities in ovulation (130), resulting in infertility in female mice (131) (2). FGF21 directly affects gonadotropin-releasing hormone (GnRH) neurons through the ERK1/2 pathway to regulate GnRH secretion, thereby inhibiting the pituitary gonadal axis and affecting FSH and LH secretion, leading to reproductive dysfunction (132). FGF21 was expressed in serum of PCOS patients and in ovarian tissues of polycystic ovary (PCO) rat model. PCOS-induced obesity and IR resulted in elevated serum FGF21 in PCOS patients. In the ovarian tissues of PCO rats, the expression of FGF21 was significantly higher than that of healthy rats, which was most pronounced in granulosa cells, probably through the up-regulation of FGF21 by PPARγ. Studies have demonstrated that FGF21 levels are increased in PCOS and are associated with HOMA-IR, BMI, body fat percentage, and WHR, regardless of Estradiol levels and FAI. This suggests that elevated FGF21 is linked to metabolic disturbances in PCOS, but not to hormonal imbalances (133).

### 2.11 SHBG

Sex hormone binding globulin (SHBG) is primarily produced and released by the liver, where it binds strongly to circulating testosterone, indicating the level of active androgens in the body (134). Low concentrations of SHBG are considered to be an independent predictor of the occurrence of metabolic diseases such as T2DM, MetS, and NAFLD. And it is also a marker of IR (135, 136). SHBG plays an important role in the pathogenesis of PCOS and is associated with its complications and long-term prognosis. Insulin increases bioactivity by inhibiting hepatic SHBG synthesis (137, 138). Thus, it can be hypothesized that low SHBG-associated NAFLD may contribute to the development of PCOS. A reduced SHBG level in NAFLD may initiate a cascade of increased androgen production, which could worsen PCOS (137). Hepatic dysfunction in NAFLD may also influence the metabolism of sex steroids, such as HA. Therefore, it can be hypothesized that low SHBG levels associated with NAFLD might contribute to the development of PCOS (139). The crosstalk role of SHBG may reflect a new liver-ovarian axis (137).

#### 2.11.1 Regulatory aspects of glucose metabolism

1. Gene polymorphisms:SHBG inherited single nucleotide polymorphisms in the human *SHBG* gene have been associated with the development of T2DM. The carriers of the SHBG rs6257 allele (CC or CT) have a higher risk of developing T2DM, whereas carriers of the rs6259 allele (AA or AG) have a lower risk of T2DM (140). And the SNPs rs1799941 in SHBG have also been associated (141).

2. Impact on glycated hemoglobin and glucose regulation: Changes in SHBG levels occur before clinical glucose abnormalities appear. SHBG levels are inversely associated with glycated hemoglobin levels, suggesting that SHBG could play a role in the early detection of individuals at risk for T2DM (142).

3. Regulation of hepatic gluconeogenesis: SHBG influences fasting blood glucose levels in humans by directly affecting hepatic gluconeogenesis (143).

#### 2.11.2 SHBG and IR

Insulin plays a key role in regulating SHBG metabolism, and SHBG is a strong indicator of insulin sensitivity. Therefore, a decrease in SHBG levels is a predictor of the development of IR (144). Hepatocyte nuclear factor 4 alpha(HNF-4α), a key transcription factor of SHBG, also activates the promoters of several genes related to lipid metabolism in the liver (145). HNF-4α is reduced in the livers of obese/IR mice (146), and the HNF-4α single nucleotide polymorphisms and haplotypes correlate with IR and T2DM risk (147). So HNF-4α may serve as a bridge to link the SHBG-IR correlation. Low SHBG levels mediate IR in several ways, such as (1) Intrahepatic fat inhibits SHBG expression and reduces hepatic insulin sensitivity by decreasing HNF-4α (148) (2), It may also mediate IR independently of intrahepatic fat (149) (3), It indirectly mediates insulin sensitivity by regulating serum sex hormone levels and inhibiting adiponectin (150) (4), It may downregulate the involvement of the PI3K/AKT pathway thereby playing a role in local and systemic IR (148).

#### 2.11.3 Regulation of lipid metabolism

SHBG levels are low in obese PCOS patients (137), and positively correlated with HDL-C levels and negatively correlated with the occurrence of MetS (151). SHBG is not only a biomarker of intrahepatic fat accumulation, but also involved in intrahepatic lipid metabolism. Low levels of SHBG exacerbate PCOS dyslipidemia (152), specifically (1) Obesity and MetS are associated with adipose tissue IR. It increases lipolysis, which in turn promotes hepatic gluconeogenesis and adipogenesis. These subsequently inhibit HNF-4α and reduces SHBG production (153) (2), Adipokines and inflammatory factors may regulate SHBG expression: leading to localized inflammation and worsening of IR (152), ①TNF-α released from hepatic adiposity impairs hepatic insulin signaling and promotes the accumulation of intrahepatic TGs, leading to the downregulation of HNF-4α mRNA (154, 155), ②The adipose inflammatory cytokine IL-1β can inhibit the expression of HNF-4α through activation of the MEK1/2 and JNK/MAPK pathways (156). Thus, adipose tissue IR down-regulates hepatic SHBG. Correspondingly, SHBG also inhibits inflammation and lipid accumulation in macrophages and adipocytes, which may be a potential protective mechanism for MetS (150). SHBG overexpression significantly reduces hepatic fat accumulation through activation of the MEK1/2 pathway. Therefore, SHBG may be a therapeutic target for NAFLD overexpression of SHBG significantly decreases hepatic fat accumulation by activating the MEK1/2 pathway. SHBG could serve as a potential therapeutic target for NAFLD (157, 158).

#### 2.11.4 SHBG and PCOS

1. IR can lead to higher levels of free or bioactive testosterone by acting on follicular membrane cells and reducing SHBG production in the liver. Low SHBG levels lead to HA, impair insulin sensitivity and exacerbate visceral obesity (12). In conclusion, SHBG, HA, and IR create a vicious cycle where IR, NAFLD, and PCOS interact with one another. Furthermore, low levels of SHBG stimulate the production of various active adipokines and inflammatory factors, such as C-reactive protein (CRP), which lead to localized inflammation and worsen IR (137). Low circulating levels of SHBG can be regarded as a biomarker for the diagnosis of IR inflammation (136). Reduced hepatic SHBG production is associated with HA in PCOS and is considered a biomarker of NAFLD in PCOS (137). The link between lower SHBG levels and a higher risk of NAFLD in adolescents with PCOS appears to support this conclusion (159).

2. HA exacerbates PCOS and HA reduces circulating adiponectin levels, a key factor in the development of IR in PCOS (160). The mechanisms involved, such as the excess of androgens, lead to adipocyte hypertrophy (161, 162), which induces IR and HI (163), or IR by reducing insulin clearance (164). HA further interferes with the negative feedback regulation of the hypothalamic-pituitary-ovarian axis, reduces the hypothalamus’ sensitivity to LH pulses, increases LH release, and raises the LH/FSH ratio (137), which further stimulates the ovaries to increase androgen secretion. They altogether form a vicious cycle.

3. Gene polymorphisms: SHBG polymorphisms with eight or more (TAAAA) n pentanucleotide repeat sequences (rs35785886) are associated with PCOS risk and low serum SHBG concentrations in PCOS (165). Another study evaluated polymorphisms in genes affecting androgen synthesis and SHBG and found that the CYP17A1 rs743572 gene polymorphism was negatively associated with susceptibility to PCOS (166).

4. Distant complications: lower SHBG levels were significantly associated with an increased risk of PCOS. PCOS with lower SHBG levels were more likely to have disease,such as HA, T2DM, IR, glucose intolerance, obesity, infertility, and cardiovascular disease (167).

### 2.12 CRP

CRP is secreted by hepatocytes in response to various inflammatory stimulition (168). Interleukin-6(IL-6) and TNF-α are key inflammatory factors that regulate CRP, primarily IL-6 (169). TNF-α also directly induces hepatic secretion of IL-6, which further regulates CRP (170). Studies have indicated that elevated CRP levels may already be present in individuals with IR, appearing before the onset of significant T2DM. Additionally, individuals with higher CRP concentrations have a greater relative risk of developing metabolic MetS (171). Thus, elevated CRP is a significant predictor of developing diabetes, independently of obesity and IR, and is also closely related to its complications (172). High levels of CRP are also a potential cause of poor long-term prognosis in PCOS (173). CRP is associated with impaired glucose tolerance, impaired fasting glucose, T2DM, IR, and the formation and progression of atherosclerosis (174–177). So it is not only a biomarker of these diseases, but also important in their development, with the possible mechanism of CRP activation of NF-κB through the RHO kinase pathway (178). The pathway leads to the development of a number of diseases, including plasminogen activator inhibitor 1, PAI-1, NO and IL-6, and transcription of various molecules that play important roles in diseases such as inflammation and MetS (179, 180).

#### 2.12.1 Regulatory aspects of glucose metabolism

1. Inhibition of GLUT4: Elevated CRP inhibits the translocation of GLUT4 to the cell membrane and mediating a decrease in glucose uptake in muscle and fat, which induce disturbances in glucose metabolism (181).

2. Regulation of leptin signaling and pro-inflammatory responses: CRP acts as a leptin-binding protein, blocking leptin signaling and generating leptin resistance, which induce glucose metabolism disorders and IR (182).

3. Inhibition of insulin signaling pathway: CRP inhibits hepatic and muscle insulin signaling through insulin receptor phosphorylation and activation of PI3K and Akt (183). As well as it activates the signaling pathway through Syk tyrosine kinase and RhoA, inducing phosphorylation of JNK and IRS-1, which inhibit endothelial cell insulin signaling (184).

4. Involvement in macrophage proliferation: CRP increases monocyte MCP-1 expression and promotes macrophage proliferation, which promote adipose tissue IR formation (183).

5. Regulation of adiponectin expression: CRP stimulates the production of TNF-α, which inhibits lipocalin (185), decreases insulin sensitivity, and reduces hepatic gluconeogenesis, thereby improving glucose homeostasis (186).

#### 2.12.2 CRP and IR

Under normal conditions, insulin inhibits hepatic synthesis of CRP. In IR, CRP is elevated. Chronic inflammation may also be a key initiator of IR (187). Both dysglycemic and dyslipidemic states can induce cellular inflammation. When inflammation occurs, adipocytes release cytokines such as IL-6 and TNF-α, leading to compensatory HI and IR. At the same time, IL-6 and TNF-α up-regulate hepatic CRP, and elevated CRP can further stimulate the release of inflammatory mediators, such as IL-6 and TNF-α, to exert an inflammatory effect. Thus, CRP, together with IL-6 and TNF-α, exert inflammatory effects and interact with IR.

#### 2.12.3 Regulation of lipid metabolism

1. Mediating foam cell formation: CRP facilitates foam cell formation by enhancing LDL uptake in macrophages and influencing the production of reactive oxygen species. Additionally, CRP promotes the transformation of macrophages into foam cells and regulates lipid metabolism, contributing to the development of atherosclerotic plaques (188).

2. Regulation of hepatic lipid metabolism: CRP influences hepatic lipid metabolism by promoting IR and enhancing the breakdown of adipose tissue, which releases free fatty acids into the liver. Furthermore, during IR, peripheral tissues experience reduced insulin sensitivity, while hepatic very low-density lipoprotein(VLDL) levels rise, contributing to disruptions in hepatic lipid metabolism (189).

3. Regulation of adiponectin expression: CRP stimulates TNF-a production and inhibits lipocalin, thereby affecting hepatic lipogenesis (190, 191).

4. Regulation of leptin signaling: Leptin specifically decreases the expression of enzymes involved in hepatic lipid synthesis, while also regulating hepatic cholesterol metabolism and lowering TG and VLDL-c levels in the liver (190). CRP functions as a leptin-binding protein and induces leptin resistance, which impacts hepatic lipid metabolism (182).

#### 2.12.4 CRP and obesity

CRP levels in obese patients were significantly higher compared to non-obese patients. CRP showed a positive correlation with BMI, WHR, and waist circumference, indicating a strong association with total body fat, particularly visceral fat. However, most obese patients had CRP levels below 10 mg/L, suggesting that obesity is a state of low-grade chronic inflammation (192). Obesity is a predominantly pro-inflammatory process, and the main mechanism is that in obesity, classically activated macrophages (M1) have higher concentrations than alternatively activated macrophages (M2), expressing pro-inflammatory cytokines, and activating pro-inflammatory pathways, such as the JNK and TNF-kB signaling pathways, which lead to low-grade chronic inflammation throughout the body (190).

#### 2.12.5 CRP and MetS

MetS is also a chronic systemic low-grade inflammatory state. Inflammation may play an important role in MetS, and CRP can be used as a predictor of MetS (193). CRP levels were positively correlated with BMI, TG, blood pressure and FPG, but negatively correlated with HDL-C (194).

#### 2.12.6 CRP and NAFLD

CRP may be a potential biomarker of NAFLD and involved in NAFLD through the following pathways (195) (1). CRP is involved in NAFLD by inhibiting the insulin signaling pathway through activation of Smad3/mTOR signaling by TGF-β and ERK/mitogen-activated protein kinase (MAPK) (195) (2). CRP affects the regulation of lipid metabolism by leptin, which restricts storage of TG to prevent lipotoxicity, exerts an inhibitory effect on adipogenesis, and increase insulin sensitivity by inhibiting hepatic glucose and fat (196). Leptin shows a dual role in the development of NAFLD, preventing early hepatic steatosis and also acting negatively as an inflammatory and fibrotic factor (197) (3). By inducing ROS production and leading to impaired mitochondrial function.CRP upregulates ROS in target cells via Fcγ receptors (198), leading to further mitochondrial dysfunction and chronic inflammation (199, 200). ROS overproduction inhibits antioxidant production, leading to further oxidative damage in NAFLD (201).

#### 2.12.7 CRP and PCOS

PCOS is a disease of severe reproductive endocrine disorders.And HA, IR, obesity and inflammation interact with each other to influence the course of PCOS. CRP is the most reliable circulating marker in chronic low-grade inflammation in PCOS (202). Systemic and localized inflammation in the ovary may be the initial pathophysiological alteration in PCOS, and these inflammatory factors work together to influence the onset and progression of PCOS (203). Inflammatory factors are significantly increased in both serum and follicular fluid in PCOS, and the possible pathways such as (1): Infiltration from the circulation through the serum-granulocyte barrier (2), Up-regulation of inflammatory mediators in the ovarian granulocytes, which leads to an increase in local infiltration of circulating increased inflammatory cells into the ovarian tissues,and within the follicles (203). The elevated levels of inflammatory factors in PCOS are not related to obesity, but obesity exacerbates inflammatory state (204, 205). Elevated CRP in PCOS may be a predictive risk factor for the development of T2DM, and obesity and IR may be an important mechanism leading to its chronic inflammation, with all three contributing to each other in a vicious circle (206).

PCOS-related hyperandrogenism (HA) could potentially contribute to inflammation in adipose tissue. The possible mechanisms include: (i) androgen-induced hypertrophy of adipocytes, resulting in tissue hypoxia or the death of enlarged adipocytes, thereby triggering an inflammatory cascade and increasing CRP level (207). (ii) HA can activate mononuclear cells (MNCs) in a fasting state, and circulating MNCs as well as MNC-derived macrophages in tissues stimulate the production of adipocytokines in a paracrine manner. These adipocytokines, in turn, trigger the release of pro-inflammatory factors like TNF-α and IL-6, resulting in an increase in CRP levels (208). In a similar manner, the local increase in TNF-α encourages the hyperplasia of ovarian granulosa cells, resulting in the production of more androgens, creating a vicious cycle that continuously stimulates subacute inflammatory responses. IR also plays a pivotal role in chronic low-grade inflammation in PCOS. IR in adipose tissue increases lipolysis, along with hypertrophy of adipocytes caused by HA. These factors lead to adipose tissue dysfunction, activation of sympathetic nerves, and the further promotion of systemic inflammatory responses through the activation of the renin-angiotensin system, which in turn leads to an elevated serum CRP as systemic inflammation (209).

Accordingly, chronic inflammatory state in women with PCOS can directly trigger HA, specifically (1) CRP induces the inflammatory response with upregulation of the ovarian steroidogenic enzyme CYP17 in response to pro-inflammatory stimulition. CYP17 is the major rate-limiting enzyme in ovarian androgen synthesis, and its upregulation promotes hyperandrogenic state (210) (2), TNF-α, a pro-inflammatory cytokine, inhibits LH-dependent androgen production (211).

## 3 Future perspectives

**Figure 3:**
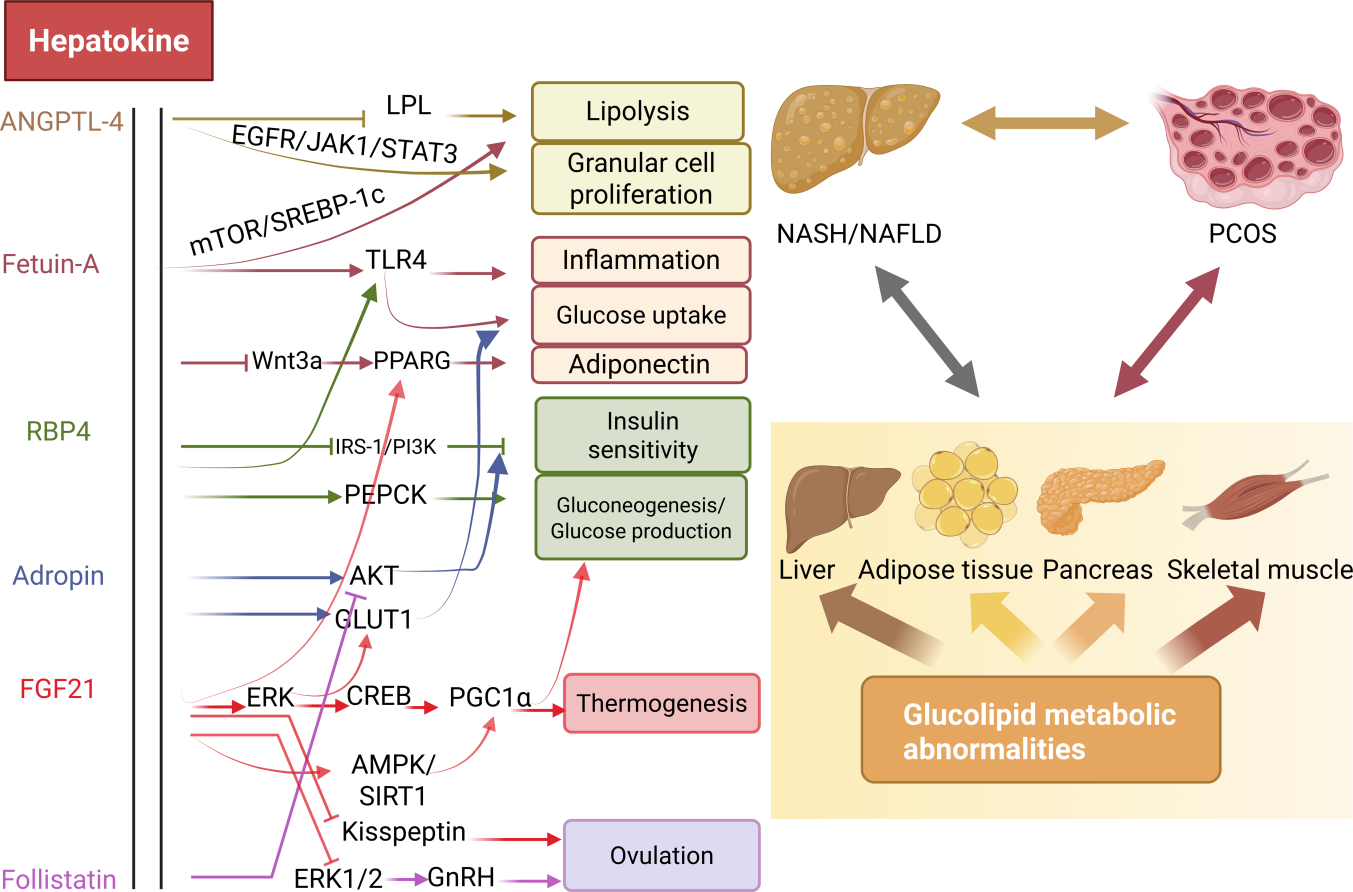
The role of hepatokines in the crosstalk of glycolipid metabolism and PCOS. Hepatokines directly affect ovarian ovulation through various signaling pathways and indirectly affect ovarian physiology through glucolipid metabolism. Hepatokines establish a link between glycolipid metabolism disorders and PCOS. The interaction of hepatokines in the progression of PCOS may reflect the liver-ovarian axis. ANGPTL-4, Angiopoietin Like 4; RBP4, Retinol-Binding Protein-4; FGF21, Fibroblast Growth Factor21; NASH, Nonalcoholic steatohepatitis; NAFLD, Nonalcoholic Fatty Liver Disease.

Different hepatokines act through distinct mechanisms within the hepato-ovarian axis to influence PCOS and its associated glucolipid metabolic disturbances. At present, the diagnosis of PCOS still relies on conventional criteria, underscoring the need for more standardized and comprehensive testing modalities. Moreover, the pathogenesis of PCOS remains incompletely understood, and current therapeutic strategies face considerable limitations. Hepatokines hold promise on multiple fronts: they may serve as candidate biomarkers for predicting disease onset and progression, offer insights into underlying mechanisms, and most crucially represent potential therapeutic targets for both PCOS and its complications. By deciphering the hepatokine-mediated communication between the liver and ovaries, clinicians and researchers can move toward more integrated approaches to women’s health. Such approaches ensure that interventions are holistically designed, aiming not only to preserve reproductive capacity and more importantly to sustain metabolic health, longevity, and quality of life. These insights not only inform clinical science but also carry implications for public health interventions and policy formulation.

(**Figure 3**. Shows the role of hepatokines in the crosstalk of glycolipid metabolism and PCOS).

(**Table 1**. Shows comprehensive overview of hepatokines).

**Table 1:** Comprehensive overview of hepatokines.

Hepatokines	Expression in PCOS and effects on PCOS	Effects on glucose and lipid metabolism *in vitro* and/or in animals	Site of action	Site of action	Correlations in humans	Prediction of the complication of PCOS in humans
ANGPTL-4	Increased Inhibit the proliferation of granule cells	Lower blood sugar Improve glucose tolerance Increase insulin sensitivity in the liver Reduce insulin sensitivity in the periphery Inhibition of the LPL activity Promote the ectopic lipid storage Increase hepatic lipid deposition Improve obesity	Liver Adipose tissue Skeletal muscle	Liver Hypothalamus Adipose tissue Ovary	T2DM Obesity Dyslipidemia NAFLD Atherosclerosis ↓	Participation in the IR of PCOS
Fetuin-A	Increased	Aggravate IR Increase hepatic and fat lipid deposition Promote qualitative changes in liver lipids Induce inflammation	Liver Adipose tissue Pancreas	Liver Adipose tissue Pancreas Skeletal muscle	Atherosclerosis CVD NAFLD MetS	Prevent PCOS
Fetuin-B	Increased	Inhibit sugar metabolism Aggravate Impaired glucose tolerance Reduce insulin sensitivity Inhibit fat synthesis Promote lipid metabolism	Liver Adipose tissue	Liver Skeletal muscle	Link NAFLD to T2DM by inducing IR	Be regarded as a candidate biomarker and therapeutic target of PCOS
Selenop	Increased	Inhibit insulin signaling	Liver Adipose tissue	Liver Brain Pancreas Skeletal muscle	NAFLD↓ MetS↓	Oxidative stress biomarkers of PCOS
RBP4	Increased	Induce inflammation in the adipose tissue Reduce insulin sensitivity Increase liver glucose	Liver Brain Adipose tissue	Liver Adipose tissue Skeletal muscle	IR Hypertriglyceridemia Atherosclerosis CVD NAFLD	Potential differential markers for hyperandrogenemia of PCOS Link the adipose tissue with the IR in PCOS A compensatory mechanism preventing the intensification of obesity-type PCOS
Adropin	Decreased	Enhance insulin signaling Improve IR Inhibition of gluconeogenesis Reduce lipid accumulation Protect the liver injury against NASH	Liver Brain Adipose tissue Pancreas Ovary	Liver Adipose tissue Skeletal muscle	Obesity↓ NAFLD↓	Affect the development of PCOS IR A strong predictor of the long-term risk in MetS in the PCOS Mediate the development of PCOS Regulate SHBG to affect hyperandrogenemia
DPP4	Increased	Inactivate the islet-high glucagon-like polypeptides Increase liver inflammation Increase liver fat Aggravate IR	Liver Brain Pancreas Skeletal muscle	Adipose tissue Skeletal muscle	IR Dyslipidemia NAFLD	Aggravate IR in PCOS
GDF-15	Decreased	Cause pancreatic islet β cell apoptosis Improve insulin sensitivity Promote fat decomposition	Liver Adipose tissue Skeletal muscle Ovary	Brain Adipose tissue Pancreas Skeletal muscle	IR↕ Diabetes mellitus ↓ Obesity↓ NASH↓ NAFLD↓ Atherosclerosis ↓	Be associated with early PCOS
IGF-1	Increased Act on the HPA axis	Increase energy consumption Enhance the glucose uptake Improve insulin sensitivity Inhibit insulin secretion Promote lipid absorption and oxidation Reduce triglycerides Reduce cholesterol Reduce fat Resist liver fibrosis	Liver Pancreas	Liver Hypothalamus Adipose tissue Skeletal muscle Ovary	IR↓ T2DM↓ Obesity↓ Dyslipidemia ↓ Atherosclerosis ↓	A key factor in the pathogenesis of PCOS
Follistatin	Increased Inhibit FSH	Promote the pancreatic islet β -cell proliferation Aggravate IR Reduce lipid accumulation Inhibit white adipose tissue decompose Increase the release of FFA	Liver Hypophysis Adipose tissue	Liver Hypophysis Adipose tissue Pancreas Skeletal muscle Ovary	IR↑ T2DM↑ Obesity ↓ Atherosclerosis ↑ NAFLD↑	Be related to the chronic low-grade inflammatory status of PCOS
FGF21	Increased	Increase glucose uptake by adipocytes Upregulate GLUT-1 expression in adipocytes Protect pancreatic islet β -cells Regulate hepatic gluconeogenesis Promote glucose uptake in skeletal muscle Reduce sugar intake Inhibit the hepatic fat synthesis Promote the oxidation of fatty acids in the liver Inhibit lipid delivery to the liver Promote lipophagy in liver adipocytes Promote browning of white adipose tissue Improve inflammation Improve oxidative stress Inhibit liver fibrosis	Liver Brain Adipose tissue Pancreas Skeletal muscle	Liver Brain Adipose tissue Pancreas Skeletal muscle oarium	IR↓ NASH↓ NAFLD↑	Be associated with a disordered PCOS metabolism
SHBG	Decreased Increase free testosterone	Improve IR Inhibit inflammation Reduce lipid accumulation Downregulate lipogenesis Regulate hepatic gluconeogenesis	Liver	Liver Adipose tissue	T2DM↓ MetS↓ NAFLD↓	A Strong predictor of insulin sensitivity A biomarker of IR inflammation Increase the risk of PCOS significantly A predictive marker of IR in obese PCOS Lead to the progression of the characteristic phenotypes of PCOS Be associated with the NAFLD and IR of PCOS
CRP	Increased A leptin-binding protein	Inhibit the insulin-signaling pathway Impaired mitochondrial function Promote the LDL uptake Aggravate inflammation	Liver Adipose tissue	Liver Adipose tissue Ovary	IR↑ T2DM↑ Dyslipidemia ↑ Obesity ↑ NAFLD↑ Atherosclerosis ↑	A significant predictor of diabetes mellitus A predictor of the MetS An inflammatory marker of PCOS

**Table 1** Symbols without asterisks indicate an association but without proven direction; ↑, promotion/upregulation; ↓, inhibition/downregulation.

## 4 Conclusion

The hepatokines influence various aspects of glycolipid metabolism disorders and play significant roles in several glycolipid metabolism-related diseases. More importantly, they impact the progression of PCOS through different pathways, establishing a link between glycolipid metabolism disorders and PCOS. This highlights the crucial role of communication between the liver and other organs in the development of PCOS, particularly the essential role of hepatokine-mediated liver-ovarian interaction in PCOS.
